# The fungicide pyraclostrobin affects gene expression by altering the DNA methylation pattern in *Magnaporthe oryzae*

**DOI:** 10.3389/fpls.2024.1391900

**Published:** 2024-04-30

**Authors:** Shumei Fang, Hanxin Wang, Kaihua Qiu, Yuanyuan Pang, Chen Li, Xilong Liang

**Affiliations:** ^1^ Heilongjiang Plant Growth Regulator Engineering Technology Research Center, College of Agriculture, Heilongjiang Bayi Agricultural University, Daqing, China; ^2^ Heilongjiang Provincial Key Laboratory of Environmental Microbiology and Recycling of Argo-Waste in Cold Region, College of Life Science and Biotechnology, Heilongjiang Bayi Agricultural University, Daqing, China

**Keywords:** *Magnaporthe oryzae*, pyraclostrobin, gene expression, cytosine methylation, adenine methylation

## Abstract

**Introduction:**

Rice blast disease caused by *Magnaporthe oryzae* has long been the main cause of rice (*Oryza sativa* L.) yield reduction worldwide. The quinone external inhibitor pyraclostrobin is widely used as a fungicide to effectively control the spread of pathogenic fungi, including *M. oryzae*. However, *M. oryzae* can develop resistance through multiple levels of mutation, such as target protein cytb mutation G143A/S, leading to a decrease in the effectiveness of the biocide after a period of application. Therefore, uncovering the possible mutational mechanisms from multiple perspectives will further provide feasible targets for drug development.

**Methods:**

In this work, we determined the gene expression changes in *M. oryzae* in response to pyraclostrobin stress and their relationship with DNA methylation by transcriptome and methylome.

**Results:**

The results showed that under pyraclostrobin treatment, endoplasmic reticulum (ER)-associated and ubiquitin-mediated proteolysis were enhanced, suggesting that more aberrant proteins may be generated that need to be cleared. DNA replication and repair processes were inhibited. Glutathione metabolism was enhanced, while lipid metabolism was impaired. The number of alternative splicing events increased. These changes may be related to the elevated methylation levels of cytosine and adenine in gene bodies. Both hypermethylation and hypomethylation of differentially methylated genes (DMGs) mainly occurred in exons and promoters. Some DMGs and differentially expressed genes (DEGs) were annotated to the same pathways by GO and KEGG, including protein processing in the ER, ubiquitin-mediated proteolysis, RNA transport and glutathione metabolism, suggesting that pyraclostrobin may affect gene expression by altering the methylation patterns of cytosine and adenine.

**Discussion:**

Our results revealed that 5mC and 6mA in the gene body are associated with gene expression and contribute to adversity adaptation in *M. oryzae*. This enriched the understanding for potential mechanism of quinone inhibitor resistance, which will facilitate the development of feasible strategies for maintaining the high efficacy of this kind of fungicide.

## Introduction

*Magnaporthe oryzae* is the main causative agent of rice blast outbreaks and has severely restricted rice yield in all rice-producing countries worldwide (Shahriar et al., 2020). Blast causes a 10-30% loss in rice yield annually, an amount that is enough to feed more than 60 million people (Sella et al., 2021). Due to the widespread distribution, destructive power and strong adaptability of rice blast fungus, various control methods, such as the use of fungicides or the cultivation of resistant varieties, have been used to eliminate this fungus. However, this pathogen rapidly develops adaptive mutations that render fungicides and resistant varieties ineffective. The “tactical” response or escape strategy of rice blast fungus has long been the focus of research efforts.

The reasons for the variation in the fungal response to adverse stress and plant host resistance have been extensively studied by various techniques, and epigenetic modifications have been shown to be involved in this process (Jeon et al., 2015; Dubey and Jeon, 2017; He et al., 2020). DNA methylation, including cytosine methylation (5mC) and adenine methylation (6mA), is a common DNA epigenetic modification that has crucial effects on gene activity (Moore et al., 2013). A study on *Metarhizium robertsii* showed that the DNA 5mC level was 0.38%-0.42%, and differential DNA methylation may contribute to the temporal and spatial regulation of gene expression and the development of mycelia and conidia in this organism (Li et al., 2017). A study on *M. oryzae* showed that 5mC accounted for 0.22% of all genomic cytosines in mycelia, which underwent global reprogramming during fungal development, and this process occurred in and around genes as well as transposable elements, contributing to the silencing of transposable elements and transcript abundance of genes (Jeon et al., 2015). In *Saccharomyces cerevisiae*, *Schizosaccharomyces pombe*, *Caenorhabditis elegans*, and *Tetrahymenather mophila*, 5mC is absent; however, 6mA is present in these organisms and is involved in the regulation of DNA replication, repair, transposition, and transcription (Hattman, 2005; Liang et al., 2018; Xiao et al., 2018a). Mondo et al. reported that 6mA is widespread in fungi, with levels as high as 2.8% in some fungi. It mainly appears symmetrically in the ApT sequence at the transcription initiation point (Mondo et al., 2017). Additional studies have shown that 6mA may be a common epigenetic marker in eukaryotes, including humans, pigs, mice, fishes, frogs, flies, worms, ciliates, Chlamydomonas and fungi, and potentially functions in transcriptional activation or silencing, chromatin regulation, and the stress response (Fu et al., 2015; Greer et al., 2015; Mondo et al., 2017; Liang et al., 2018; Xiao et al., 2018b).

Although much progress has been made in understanding the adaptation of *M. oryzae* to fungicides (Kim and Kim, 2009; Bohnert et al., 2019; Xu et al., 2020; Zhang et al., 2020), the contribution of epigenetic factors, such as DNA methylation, to this enigmatic process remains to be studied. As shown by Jeon et al. (2015), 5mC could be a dynamic epigenetic factor contributing to fungal development and genome defense in *M. oryzae*. In this work, we evaluated the gene expression response of *M. oryzae* to the fungicide pyraclostrobin and its relationship to changes in 5mC and 6mA methylation.

Pyraclostrobin is an exoquinone inhibitor that can bind to ubiquinone site of the cytochrome bc1 complex to prevent electron transport on the inner mitochondrial membrane. This interferes with the process of oxidative phosphorylation, resulting in insufficient production of cellular energy ATP and ultimately leading to fungal death. *M. oryzae* strain is sensitive to pyraclostrobin, with half maximal effective concentration (EC50) of 0.0012-0.0128 μg/mL (Ruan et al., 2022a). The sensibility was higher than other popular fungicides such as tebuconazole, carbendazim, propiconazole (Ruan et al., 2022a). However, the rapid development of resistance in *M. oryzae* to the fungicide and consequent control failure has become increasingly problematic. The main mechanism conferring resistance involves mutations in the cytochrome b gene *CYTB*, causing the substitution of glycine by alanine or serine at position 143 (G143A/S) (Li et al., 2022; Peng et al., 2022; Ruan et al., 2022b). The overexpression of alternative oxidase gene (*AOX*) in the alternative oxidation pathway and efflux transporter ATP-binding cassette (ABC) gene *MoABC-R1* are also likely to contribute to the resistance (Gisi et al., 2002; Fernández-Ortuño et al., 2008; Hu et al., 2023). This study will enhance the understanding for potential mechanism of quinone inhibitor resistance, which will facilitate the development of drug targets as well as feasible strategies for keeping the high efficacy of this kind of fungicide.

## Methods

### Strain culture conditions and pyraclostrobin treatment

*M. oryzae* Y34 stored on paper filters at -20°C in our laboratory was cultured at 28°C on complete agar media for assessment of growth traits. The mycelia used for DNA and RNA extraction were cultured in 250 mL of liquid CM (1 g/L yeast extract, 0.5 g/L casein enzymatic hydrolysate, 0.5 g/L casein acid hydrolysate, 10 g/L glucose, 1 g/L Ca(NO_3_)_2_·4H_2_O, 0.2 g/L KH_2_PO_4_, 0.25 g/L MgSO_4_·7H_2_O, 0.15 g/L NaCl) at 28°C and 150 rpm. After three days, pyraclostrobin (CAS:175013-18-0) was added, and the final concentration reached 7.4 µg/L (the EC50 data are shown in **Supplementary Table 1**). The control group was treated with the same volume of sterile water. Each treatment was repeated three times. After 24 hours, the mycelia were collected by filtration and washed three times with deionized water. The collection in each flask was divided equally into 2 replicates for DNA and RNA extraction.

### Transcriptome and methylome sequencing

**Figure 1:**
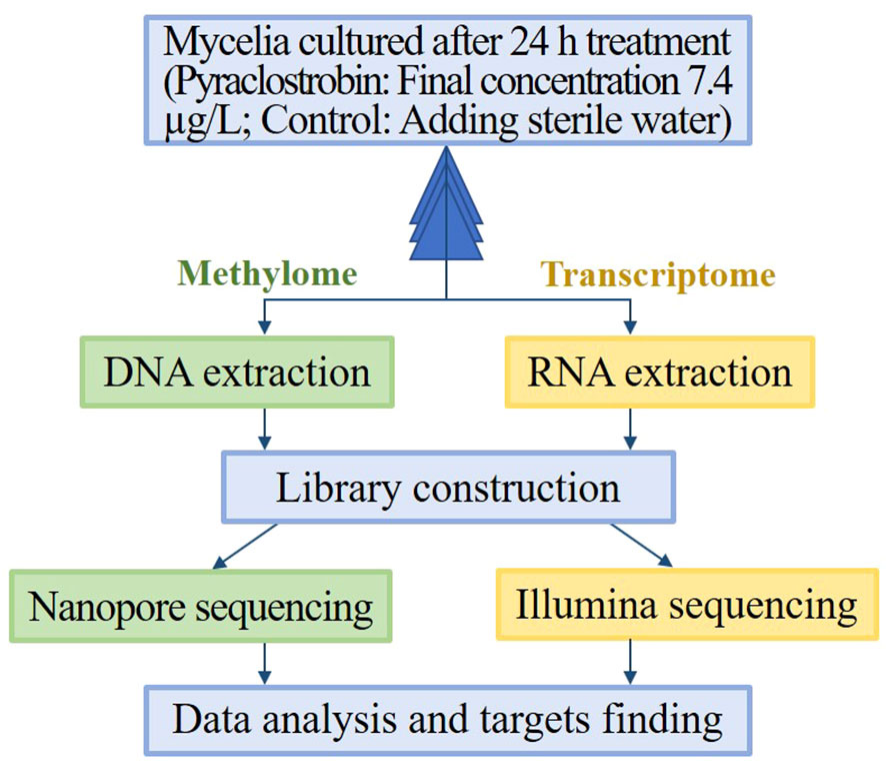
Schematic overview of the methyl-seq and RNA-seq protocol. Mycelia cultured after 24 h treatment (Final concentration of pyraclostrobin was 7.4 µg/L, and control was added the same volume of sterile water) were collected from each flask and were divided into two parts for DNA extraction and RNA extraction. After nanopore sequencing and Illumina sequencing, data analysis was performed to obtain differentially methylated genes and differentially expressed genes, and then possible metabolite process was analyzed by both GO and KEGG pathway.

As shown in **Figure 1**, The mycelia for transcriptome and methylome sequencing were cultured from the same batch of flasks. The collection from each flask was divided into two parts with three biological replicates per treatment. The mycelia collected were frozen in liquid nitrogen and stored at -80°C until DNA and RNA isolation. Total RNA was extracted using a TRIzol kit (Invitrogen, CA, USA), and mRNA was enriched with oligo-dT primers. A cDNA library was generated for transcriptome sequencing on the HiSeq X Ten platform. The nanopore sequencing method was applied to detect methylated bases in genomic DNA; this method is reported to be sensitive enough to detect chemical modifications on genomic DNA (Rand et al., 2017; Simpson et al., 2017). A nanopore library was constructed according to the protocol provided by Oxford Nanopore Technologies as follows: Genomic DNA was extracted; the DNA concentration was quantified using a Nanodrop spectrophotometer and a Qubit fluorometer, and DNA integrity was detected by 0.35% agarose gel electrophoresis. Genomic DNA was fragmented into 8 kb fragments using a gTube. Library construction was performed using the SQK-LSK109 kit (Oxford Nanopore Technologies, Oxford, UK). All library preparation and sequencing were conducted by Wuhan Benagen Technology Company Limited (Wuhan, China). The raw data has been deposited in NCBI (BioProject accession number PRJNA1096937).

### Gene expression and functional enrichment analysis of DEGs

After quality assessment and data filtration, clean reads were obtained. Multiple sequence alignments of DNA were performed using Star 2.7.0d software (Dobin et al., 2012). The quality control and data management software used was QoRTs (Hartley and Mullikin, 2015). HTSeq software (Trapnell et al., 2010) was used to obtain the number of reads aligned to each gene for each sample. The quantitative results are expressed as FPKM values (expected number of fragments per kilobase of transcript sequence per millions base pairs sequenced) corrected by TMM (EdgeR’s trimmed mean of M values). Based on the expression levels of all genes in each sample, differential expression analysis was completed by DESeq2 (Anders and Huber, 2010). The filtration thresholds were q value<0.05 and |log2FoldChange|>1. Functional enrichment analysis of differentially expressed genes (DEGs) was performed using clusterProfiler (Yu et al., 2012), including Gene Ontology (GO) and Kyoto Encyclopedia of Genes and Genomes (KEGG) pathway analyses. GO enrichment analysis can provide molecular function information on DEGs. KEGG pathway analysis was used to analyze the biochemical metabolic processes. P values ≤ 0.05 were considered to indicate significant enrichment.

### DNA methylation analysis

The original ionic current signal was base called by Guppy software (Wick et al., 2019) to obtain the raw reads. The raw reads were filtered by removing low-quality reads and adaptor sequences. Then, the clean and high-quality reads were aligned to the *M. oryzae* reference genome (http://fungi.ensembl.org/Magnaporthe_oryzae/Info/Index) (Dean et al., 2005) using minimap2 software (Li, 2018). The mapping ratios were determined, and the matched reads were used for subsequent methylation analysis. The Tombo program (Stoiber et al., 2017) was used to associate the raw ionic current signals with specific genomic bases, and the 5mC, mCpG and 6mA sites were identified. RepeatMasker (Chen, 2004) was used to identify repetitive elements in the genomic sequence; the repeat regions, upstream 2 kb region, and downstream 2 kb region were divided into 50 bins, and the average methylation level of each bin was evaluated. Based on the differentially methylated sites (DMSs) identified by MethylKit (Akalin et al., 2012), the genome was segmented and then divided into differentially methylated regions (DMRs), which were analyzed using MethCP (Gong and Purdom, 2020). Fisher’s test was used to test the differences in regions, and the significance threshold was 0.01. Genes containing DMRs in exons, introns, 2 kb regions upstream of translation start sites or 2 kb regions downstream of transcription termination sites were considered differentially methylated genes (DMGs).

## Results

### Methylomic and transcriptomic features

The data of the methylome and transcriptome are shown in **Table 1**. We obtained an average of 1,293,690 and 65,179,085 clean reads from the *M. oryzae* methylome and transcriptome, respectively. All our results were obtained from these filtered reads, of which 1,160,010 and 61,878,247 reads were mapped uniquely to the reference genome (http://fungi.ensembl.org/Magnaporthe_oryzae/Info/Index) (Dean et al., 2005). The proportion of both exceeded 89.64%.

**Table 1:** Overview of the methylome and transcriptome data of *Magnaporthe oryzae*.

	Sample name	Clean reads	Unique mapped reads	Ratio /%	Depth /%	GC content/%
**Methylome**	Control	327608	297174	90.73	78.76	–
	Pyraclostrobin	472599	423024	89.64	82.00	–
	Sum	1293690	1160010	–	–	–
**Transcriptome**	Control	23056733	21857783	94.80	–	56.09
	Pyraclostrobin	20390357	19394627	95.12	–	56.15
	Sum	65179085	61878247	–	–	–

**Figure 2:**
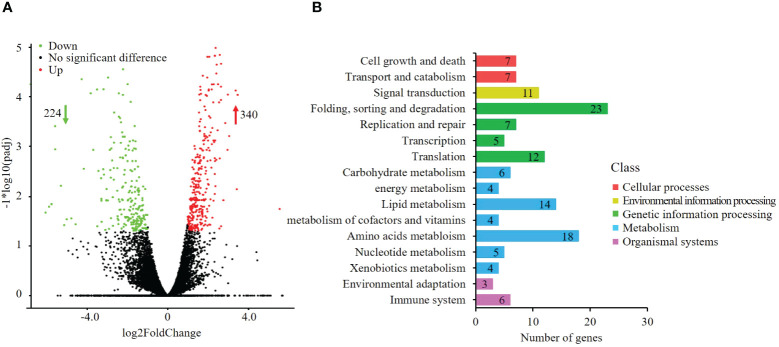
Gene expression changes and differentially expressed genes (DEGs) involved in metabolic processes under pyraclostrobin stress. **(A)** Volcano plot of differentially expressed genes after pyraclostrobin treatment; **(B)** KEGG pathway analysis of differentially expressed genes. Among the 564 (DEGs) detected, 340 genes were upregulated, and 224 genes were downregulated. These genes were clustered into five main classes: metabolism, genetic information processing, environmental information processing, cellular processes, and organismal systems.

Pyraclostrobin treatment led to the differential expression of 564 genes. Among these genes, 340 were upregulated, and 224 were downregulated (**Figure 2A**). KEGG pathway analysis revealed that these alterations mainly affected pathways associated with metabolism, genetic information processing, environmental information processing, cellular processes, and organismal systems (**Figure 2B**), such as protein processing in the ER (ko04141), ubiquitin-mediated proteolysis (ko04120), glutathione metabolism (ko00480), fatty acid metabolism (ko01212) and aminoacyl-tRNA biosynthesis (ko00970).

### Protein processing in the ER and ubiquitin-mediated proteolysis were enhanced

**Figure 3:**
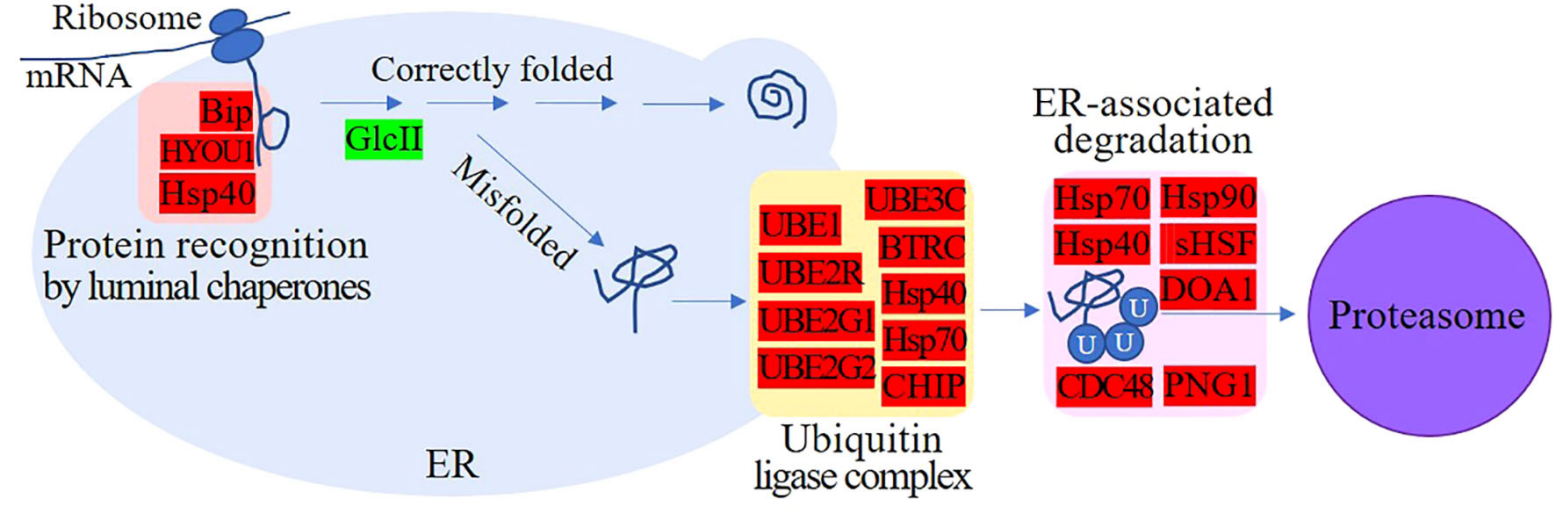
Endoplasmic reticulum-associated and ubiquitin-mediated proteolysis. Red indicates upregulated proteins; green indicates downregulated proteins. Multiple molecular chaperones that can recognize and bind nascent peptides, proteins involved in the formation of ubiquitin ligase complexes, and proteins that facilitate the entry of ubiquitin-binding proteins into the proteasome were upregulated, but proteins involved in the correct folding of glycoproteins were downregulated.

As shown in **Table 2**, multiple DEGs encoding molecular chaperones in the endoplasmic reticulum (ER), such as BiP, HYOU1, and Hsp40, which recognize and bind nascent peptides during the transport of functional proteins, were upregulated 1.20- to 2.60-fold. In addition, the proteins UBE2G1, UBE2G2, HSP40, HSP70, and CHIP, which are involved in the formation of the ubiquitin ligase complex, and the proteins sHSF, p97, HSP90, DOA1, PNG1, UBE1, BTRC, UBE3C, UBE2R, and MGG_05584, which facilitate the entry of ubiquitin-binding proteins into the proteasome, were also upregulated. These proteins jointly participate in ER-associated and ubiquitin-mediated proteolysis (**Figure 3**). The above results suggest that many polypeptides may not be able to form functional proteins with correct spatial conformations in the ER and may not directly enter the degradation program. For example, the expression of GluII, a protein involved in the folding of glycoproteins in the ER (Wilkinson et al., 2006), was downregulated, suggesting that more misfolded proteins may be produced.

**Table 2:** Differentially expressed genes involved in protein processing in the endoplasmic reticulum and ubiquitin mediated proteolysis.

Involved processes	Protein name	Gene name	Annotation	log2Fold Change	P adj
Protein processing in ER	sHSF	*MGG_04358*	Belongs to HSP20 family	2.60	0.0018
	HYOU1	*MGG_06648*	Hypoxia up-regulated protein 1	2.20	7.24E-05
	Hsp90	*MGG_06759*	Heat shock protein 90 homolog	2.15	0.0005
	DOA1	*MGG_14014*	Ubiquitin homeostasis protein lub1	2.12	1.20E-07
	BIP	*MGG_02503*	Endoplasmic reticulum chaperone BiP	1.96	3.14E-06
	Hsp40	*MGG_04462*	Mitochondrial protein import protein mas5	1.93	6.20E-07
	Hsp70	*MGG_06958*	Heat shock 70 kDa protein	1.68	0.0203
	UBE2G1	*MGG_14071*	Ubiquitin-conjugating enzyme E2 15	1.63	0.0019
	CDC48	*MGG_05193*	Cell division control protein 48	1.48	0.0068
	UBE2G2	*MGG_04081*	Ubiquitin-conjugating enzyme E2-18 kDa	1.28	0.0215
	CHIP	*MGG_08035*	Peptidylprolyl isomerase	1.23	0.0164
	PNG1	*MGG_03598*	Protein PNG1	1.20	0.0158
	GlcII	*MGG_08623*	Glucosidase 2 subunit alpha	-1.25	0.0121
Ubiquitin mediated proteolysis	—	*MGG_05584*	Ubiquitin-dependent protein catabolic process	2.01	0.0057
	UBE1	*MGG_01409*	Ubiquitin-activating enzyme E1 1	1.88	0.0007
	BTRC	*MGG_00261*	Beta-TrCP	1.30	0.0029
	UBE3C	*MGG_09504*	Probable E3 ubiquitin protein ligase C167.07c	1.25	0.0086
	UBE2R	*MGG_14266*	Ubiquitin-conjugating enzyme E2-34 kDa	1.03	0.0270

In addition, a tripeptidyl-peptidase (TPP1, MGG_07404) located in lysosomes that can degrade aging and abnormal proteins was found to be upregulated 2.58-fold. This suggests that in response to pyraclostrobin stress, *M. oryzae* may also prevent cell damage caused by abnormal protein accumulation by enhancing the lysosomal degradation pathway. Additionally, a 41 kDa peptidyl-prolyl cis-trans isomerase (PPID, MGG_08104) was upregulated 3.05-fold. PPID can accelerate the isomerization of proline peptide bonds that assist protein folding (Keogh et al., 2019). Taken together, these results show that the post-translation stage is an important stage for the response of *M. oryzae* to pyraclostrobin stress.

### The fidelity of genetic information transmission was altered

The fidelity of genetic information transmission was differentially affected by pyraclostrobin at the DNA, RNA and protein levels (**Table 3**). Multiple enzymes involved in DNA replication and repair pathways, such as DNA polymerase subunit (POLE1), DNA ligase (DNL4), DNA helicase subunit (KU70), and serine/threonine-protein kinase (TEL1), and some related proteins, such as MCM7, SLD2, TOF1, RAD5, and ER6L2, were downregulated 1.53- to 2.82-fold, suggesting that the fungicide pyraclostrobin can inhibit DNA replication and DNA repair processes, which may further affect cell division and proliferation and may result in the generation of structurally abnormal proteins. At the RNA level, the RNA polymerase subunit (ABC4) was upregulated 1.60-fold, while the changes in transcription factors were inconsistent; some transcription factors were upregulated (FAP1H, MGG_13927, MGG_00672, MNAT1, TFIIE1), while others were downregulated (ZNFX1, MGG_04674, HAP3). Some pre-mRNA splicing factors were upregulated, and most of the protein factors involved in mRNA stability and degradation were also upregulated, indicating that the drug not only did not obviously inhibit mRNA expression but also produced more protein to promote the splicing of pre-mRNA and enhance its stability. The expression levels of various aminoacyl-tRNA synthetases, ribosomal synthases and transporters that contribute to protein synthesis were all upregulated. The above results suggest that both the transcription and translation processes were enhanced. However, due to the inhibition of DNA replication and repair processes, genetic information is damaged. Although the information encoded by DNA can be faithfully passed on to mRNAs and proteins, more abnormal proteins are still produced. These abnormal proteins needed to be degraded, and thus, the proteolytic capacity needed to be enhanced, which was consistent with the aforementioned enhancement of the proteolytic process (**Table 2**).

**Table 3:** Differentially methylated genes involved in the high-fidelity transmission of genetic information within KEGG Ontology annotations in *M. oryzae*.

Levels	Process involved	Protein name	Gene name	Annotation	log2FC	P adj
DNA level	Replication and repair	RAD5	*MGG_12155*	DNA repair protein RAD5	-2.82	0.0007
		POLE1	*MGG_03850*	DNA polymerase ϵ catalytic subunit A	-2.36	0.0017
		MCM7	*MGG_09300*	DNA replication licensing factor mcm7	-2.17	0.0186
		SLD2	*MGG_11473*	DNA replication regulator SLD2	-2.16	0.0363
		KU70	*MGG_01512*	ATP-dependent DNA helicase II subunit 1	-2.09	0.0015
		TOF1	*MGG_03991*	Topoisomerase 1-associated factor 1	-1.96	0.0118
		DNL4	*MGG_10627*	DNA ligase 4	-1.91	0.0278
		TEL1	*MGG_14764*	Serine/threonine-protein kinase tel1	-1.60	0.0008
		ER6L2	*MGG_06945*	DNA excision repair protein ERCC-6-like 2	-1.53	0.0449
RNA level	Transcription	FAP1H	*MGG_00137*	FKBP12-associated protein 1 homolog, DNA-binding transcription factor activity	2.08	1.60E-05
		–	*MGG_13927*	DNA-binding transcription factor activity, RNA polymerase II-specific	1.88	0.0252
		–	*MGG_00672*	DNA-binding transcription factor activity, RNA polymerase II-specific	1.84	0.0002
		ABC4	*MGG_11667*	RNA pol I, II, and III subunit RPABC4	1.60	0.0003
		MNAT1	*MGG_03605*	RNA pol II transcription factor B subunit 3	1.35	0.0021
		TFIIE1	*MGG_06909*	Transcription initiation factor IIE subunit alpha	1.04	0.0405
		ZNFX1	*MGG_11779*	NFX1-type zinc finger-containing protein 1, DNA-binding transcription factor activity	-2.37	0.0003
		–	*MGG_04674*	DNA-binding transcription factor activity, RNA polymerase II-specific	-1.74	0.0265
		HAP3	*MGG_01653*	Transcriptional activator HAP3	-1.60	0.0112
	Pre-mRNA splicing	CWC24	*MGG_17908*	Pre-mRNA-splicing factor cwc24	1.98	1.83E-06
		U2AF1	*MGG_09948*	Splicing factor U2AF 23 kDa subunit	1.75	0.0190
		HSP70	*MGG_06958*	Heat shock 70 kDa protein	1.68	0.0203
	mRNA stability and degradation	GroEL	*MGG_03165*	Heat shock protein 60	2.11	7.06E-06
		–	*MGG_09923*	Negative regulation of mRNA polyadenylation	1.77	0.0008
		DnaK	*MGG_04191*	Hsp70-like protein	1.30	0.0065
		PABPC	*MGG_09505*	Polyadenylate-binding protein	1.10	0.0461
		PAN2	*MGG_17449*	PAN2-PAN3 deadenylation complex catalytic subunit	-1.19	0.0445
Protein level	Aminoacyl- tRNA biosynthesis	–	*MGG_08103*	Alanine-tRNA ligase	2.66	0.0001
		YARS	*MGG_02449*	Tyrosine-tRNA ligase	1.82	0.0123
		NARS	*MGG_08897*	Asparagine-tRNA ligase	1.65	0.0374
		AARS	*MGG_03607*	Alanine-tRNA ligase	1.61	0.0005
		VARS	*MGG_04396*	Valine-tRNA ligase	1.19	0.0190
		LARS	*MGG_04042*	Leucine-tRNA ligase	-1.53	0.0319
	Ribosome biogenesis and translation	NMD3	*MGG_05817*	60S ribosomal export protein NMD3	2.73	9.99E-08
		SQT1	*MGG_03080*	Ribosome assembly protein SQT1	2.64	7.06E-06
		EF1B	*MGG_04436*	Elongation factor 1-beta	2.09	0.0006
		REI1	*MGG_02505*	Cytoplasmic 60S subunit biogenesis factor REI1 homolog	1.90	0.0006
		EIF2S1	*MGG_01592*	Translation initiation factor 2 subunit 1	1.83	0.0008
		LSG1	*MGG_07525*	Large subunit GTPase 1	1.79	0.0017
		LTV1	*MGG_07524*	Ribosomal small subunit biogenesis	1.74	0.0012
		RQC2	*MGG_02697*	Ribosome quality control complex subunit 2	1.65	0.0037
		MAK16	*MGG_17694*	Ribosomal large subunit biogenesis	1.61	0.0005
		EIF6	*MGG_01671*	Translation initiation factor 6	1.36	0.0019

### Glutathione metabolism was enhanced

**Figure 4:**
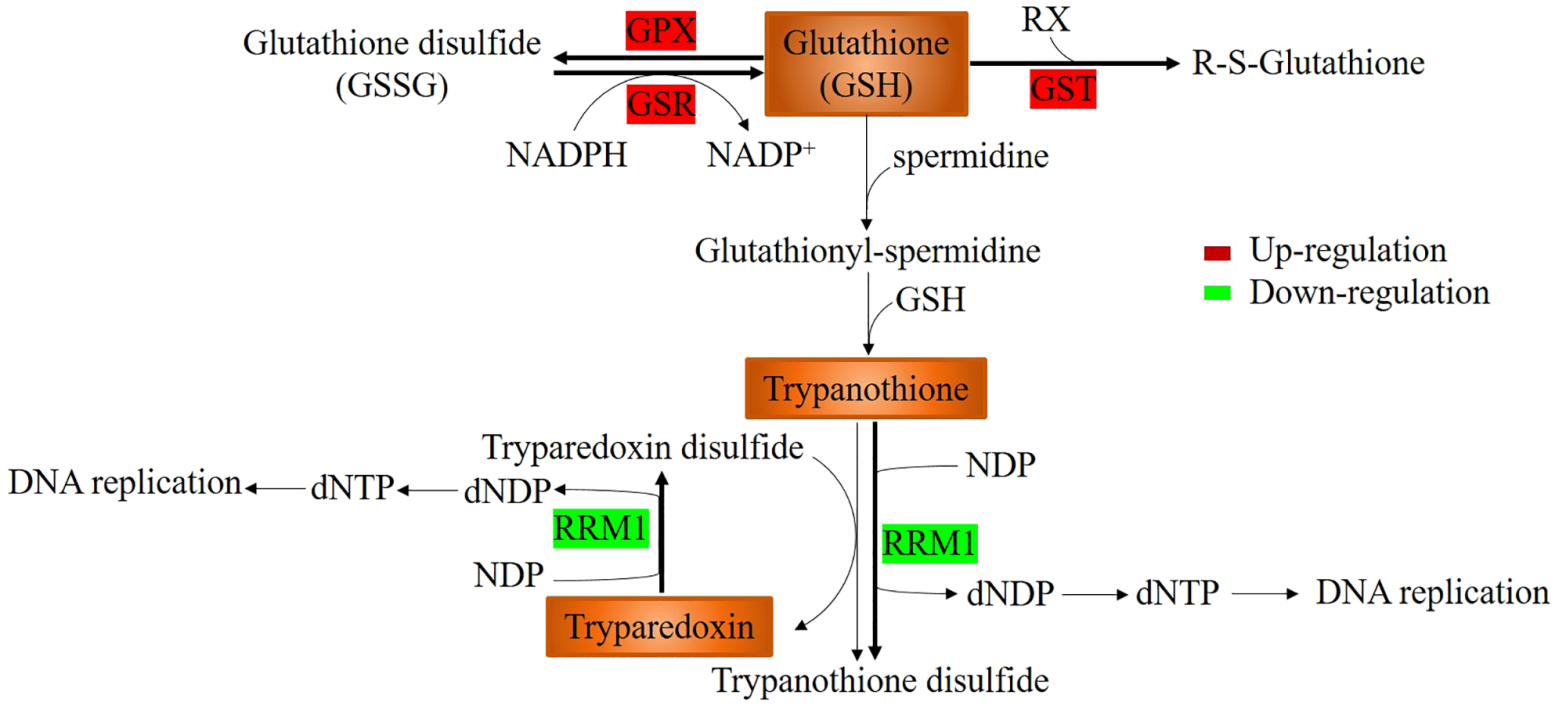
Glutathione metabolism and generation of trypanothione and tryparedoxin. Glutathione S-transferase (GST), glutathione reductase (GSR) and glutathione peroxidase (GPX) were upregulated and contributed to the removal of hydrogen peroxide and other toxic substances. Ribonucleoside-diphosphate reductase M1 (RRM1) was downregulated, which contributed to high concentrations of trypanothione and tryparedoxin.

As shown in **Table 4**, KEGG annotation indicated that the expression of enzymes related to glutathione metabolism (ko00480), including glutathione S-transferase (GST and GTO2), glutathione reductase (GSR) and glutathione peroxidase (GPX), which contribute to the removal of hydrogen peroxide and other toxic substances, and maintain a high reduced glutathione concentration, was upregulated by pyraclostrobin. This greatly protects cell tissues and sulfhydryl-containing enzymes from peroxide damage. In addition, ribonucleoside-diphosphate reductase M1 (RRM1), which is a subunit of ribonucleotide reductase, was downregulated. Ribonucleotide reductase catalyzes the biosynthesis of deoxyribonucleotides from the corresponding ribonucleotides using thioredoxin, including tryparedoxin and trypanothione, as H donors (**Figure 4**). The downregulation of *RRM1* expression contributes to high concentrations of trypanothione and tryparedoxin.

**Table 4:** Glutathione metabolism by KEGG Ontology annotation in *M. oryzae*.

Protein name	Gene name	Annotation	log2FoldChange	P adj
GTO2	*MGG_01410*	Glutathione S-transferase omega-like 2	2.87	0.0003
GST	*MGG_06747*	Glutathione S-transferase	1.72	0.0206
GSR	*MGG_12749*	Glutathione reductase	1.70	0.0026
GST	*MGG_06907*	glutathione S-transferase	1.40	0.0049
GPX	*MGG_07460*	glutathione peroxidase	1.01	0.0446
RRM1	*MGG_07000*	ribonucleoside-diphosphate reductase subunit M1	-1.54	0.0049

### Lipid metabolism was impaired

Fatty acid anabolism (ko01212) is inhibited by pyraclostrobin. As shown in **Table 5**, the genes encoding vital enzymes for the synthesis of hexadecanoyl-CoA and stearoyl-CoA, including acetyl-CoA carboxylase (ACACA), fatty acid synthase subunit α (FAS2), fatty acid synthase subunit β (FAS1), enoy-[acyl-carrier-protein] reductase (MECR) and 3-oxoacyl-[acyl-carrier-protein] reductase (FABG), were all downregulated in the presence of pyraclostrobin. Pyraclostrobin prevents electron transfer between cytochrome b and c1, inhibiting ATP production. Fatty acid synthesis is an energy-consuming process that requires large amounts of ATP and NADPH. Inhibition of fatty acid synthesis can reduce ATP consumption, which is beneficial for the synthesis of substances urgently needed for survival, such as many enzymes and proteins. This is a viable strategy that allows organisms to adapt to harsh environments.

**Table 5:** Glycerophospholipid metabolism by KEGG Ontology annotation in *M. oryzae*.

Process involved	Protein name	Gene name	Annotation	log2Fold Change	P adj
Fatty acid metabolism	FAS1	*MGG_04118*	Fatty acid synthase subunit β	-2.09	0.0054
	ACACA	*MGG_07613*	AcetylCoA-carboxylase	-1.93	0.0012
	FAS2	*MGG_12154*	Fatty acid synthase subunit α	-1.58	0.0482
	MECR	*MGG_02566*	Enoy-[acyl-carrier-protein] reductase	-1.19	0.0285
	FABG	*MGG_06660*	3-oxoacyl-[acyl-carrier-protein] reductase, or beta-ketoacyl-ACP reductase	-1.00	0.0451
Glycerophospholipid metabolism	AYR1	*MGG_16186*	NADPH-dependent 1-acyldihydroxyacetone phosphate reductase	-2.95	6.10E-10
	PLD1	*MGG_05804*	Phospholipase D1	-1.57	0.0405
	CRLS	*MGG_08851*	Cardiolipin synthase	-1.07	0.0442

In the glycerophospholipid metabolism pathway (ko00564), the expression of the *AYR1*, *PLD1* and *CRLS* genes was inhibited. AYR1 catalyzes the conversion of 1-acyldihydroxyacetone phosphate to 1-acylglycerol-3-phosphate, which is further converted to phosphatidic acid, a precursor in cardiolipin synthesis. CRLS catalyzes the conversion of phosphatidylglycerol to cardiolipin, while PLD1 promotes the breakdown of cardiolipin to phosphatidic acid.

### Additional alternative splicing events were discovered

**Figure 5:**
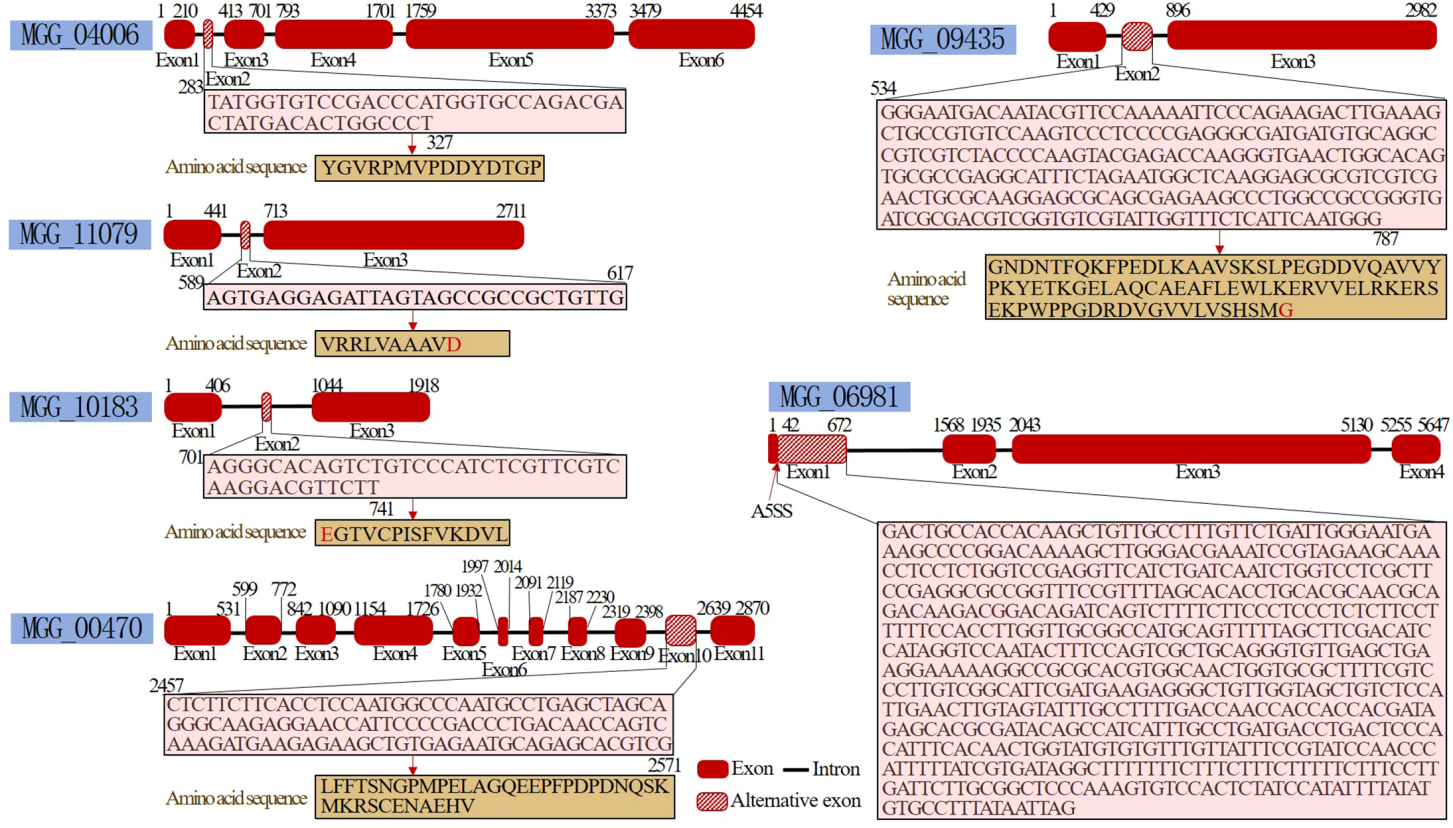
Alternative splicing after pyraclostrobin treatment. The *MGG_00470*, *MGG_09435*, *MGG_11079*, *MGG_10183* and *MGG_04006* genes exhibited exon inclusion. *MGG_06981* exhibited an alternative 5′ splice site.

After pyraclostrobin treatment, some alternative splicing events were detected, including skipped exons and alternative 5′ splice sites (**Figure 5**). Five genes, namely, *MGG_00470* (Mei2), *MGG_09435*, *MGG_11079* (nonselective cation channel), *MGG_10183* (Enolase-phosphatase E1, *UTR4*) and *MGG_04006* (Rho-GAP domain-containing protein), exhibit exon inclusion. Only the *MGG_06981* gene (carnitine O-acetyltransferase) exhibited an alternative 5′ splice site. Notably, the meiosis-related gene *MGG_00470* (Mei2) also showed a 2.27-fold decrease in gene expression according to the transcriptome sequencing results, which may be associated with its alternative splicing. Two other meiosis-related genes, *mcm7* (MGG_09300, DNA replication licensing factor mcm7) and *ANAPC1* (MGG_03314, anaphase-promoting complex subunit 1), were downregulated 2.17-fold and 1.46-fold, respectively. These results suggested that pyraclostrobin may disrupt the progression of the cell cycle.

### DNA methylation levels in the gene body and flanking regions were increased by pyraclostrobin

**Figure 6:**
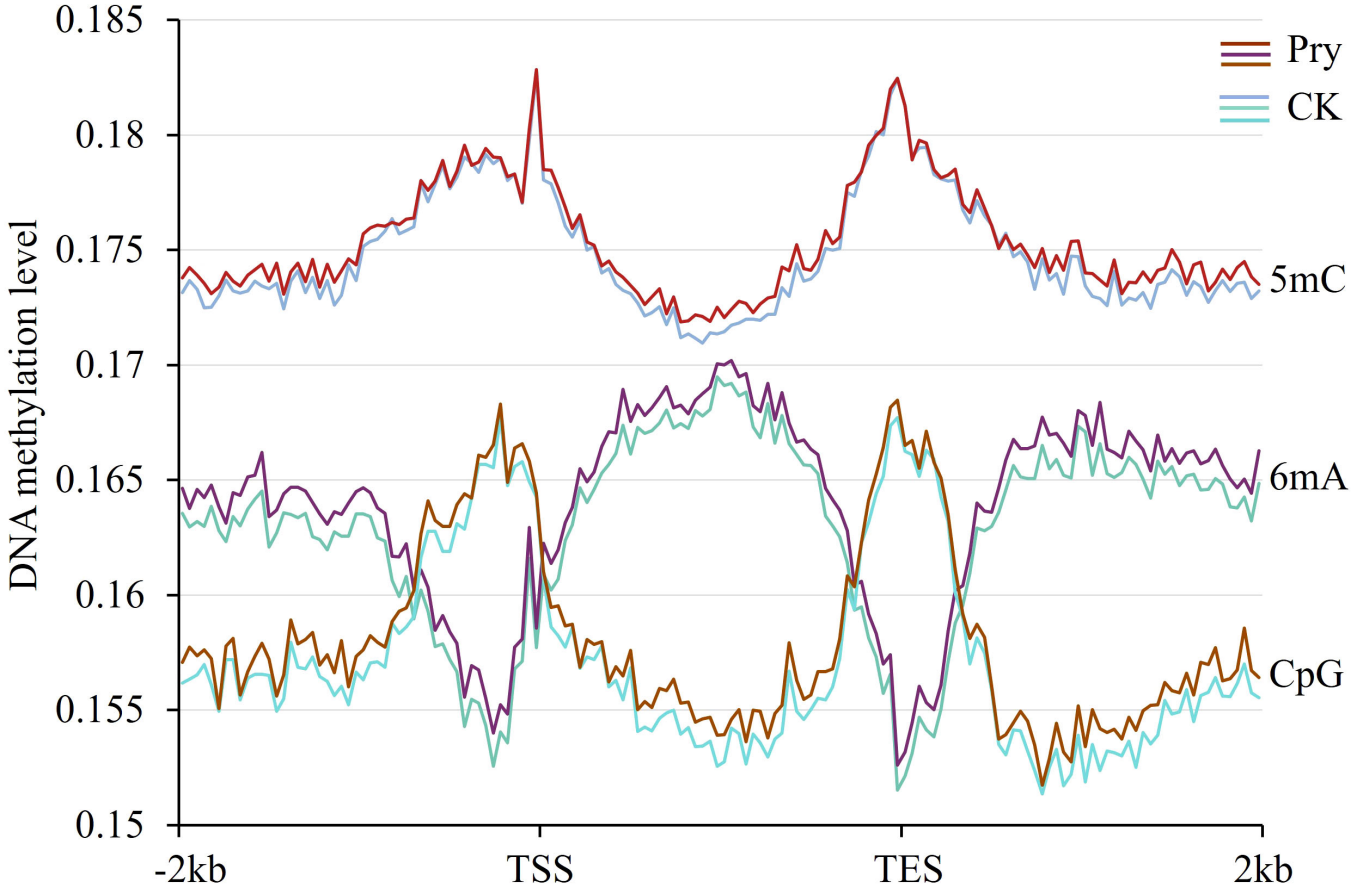
DNA methylation levels in gene regions and their changes after pyraclostrobin treatment. Pyr, pyraclostrobin; CK, control. TSS, transcriptional start site; TES, transcriptional end site. The m5C level was greater than the m6A level. At the transcriptional start site and transcriptional end site, the m5C level was high, while the m6A level was low.

To examine whether gene expression and alternative splicing are associated with DNA methylation in the gene body and upstream and downstream regions, in this work, we measured the methylation levels of 5mC and 6mA in the gene body and 2 kb upstream and 2 kb downstream flanking regions. As shown in **Figure 6**, the 5mC level was greater than the 6mA level. The methylation levels of 5mC at the transcriptional start site and transcriptional end site were significantly greater than those in other regions, which was consistent with the distribution of CpG. In contrast to the distribution of 5mC, the level of 6mA was high in the gene body region but low at the transcriptional start site and transcriptional end site. After treatment with pyraclostrobin, both 5mC and 6mA levels increased throughout the gene body and flanking regions.

### GO and KEGG enrichment analysis of DMGs

**Figure 7:**
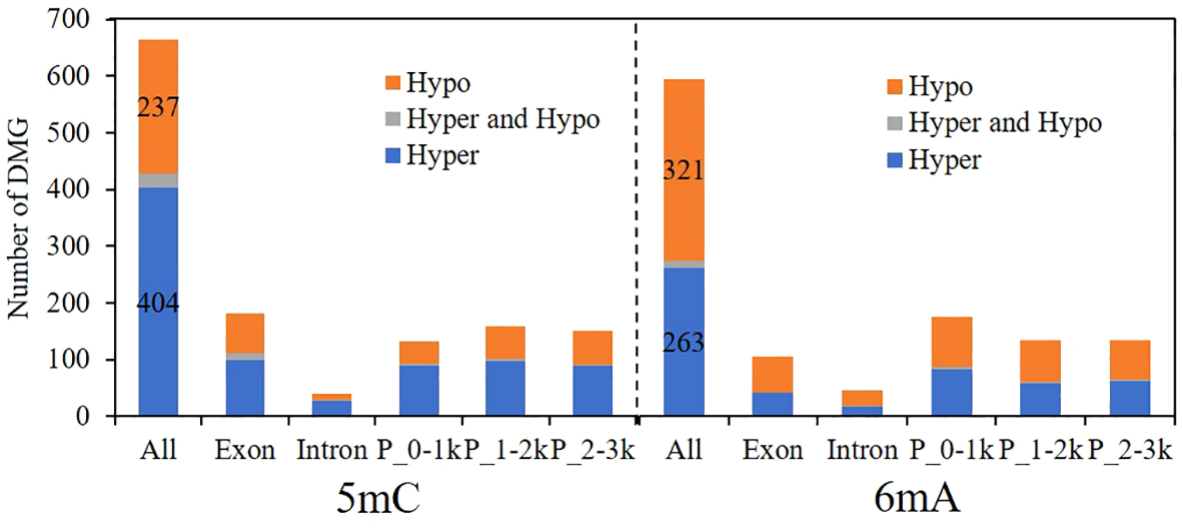
Changes in the differential methylation of genes caused by pyraclostrobin. P_0-1k, promoter_0-1 kb; P_1-2k, promoter_1-2 kb; P_2-3k, promoter_2-3 kb; Hypo, hypomethylation; Hyper, hypermethylation.

**Figure 7** shows the genes with differential methylation of 5mC and 6mA. A total of 665 genes were differentially methylated with 5mC, 404 of which were hypermethylated, accounting for 60.75%, and 237 genes were demethylated, accounting for 35.64%. A total of 596 genes exhibited differential methylation of 6mA, of which 263 genes were hypermethylated, accounting for 44.13%, and 321 genes were demethylated, accounting for 53.86%. The results showed that when the fungus responds to pyraclostrobin, 5mC is mainly hypermethylated, while 6mA is mainly demethylated, both of which occur mainly in exons and promoters, suggesting that methylation changes occurring in exon and promoter regions are probably critical for regulating gene expression to prevent damage under stress.

**Figure 8:**
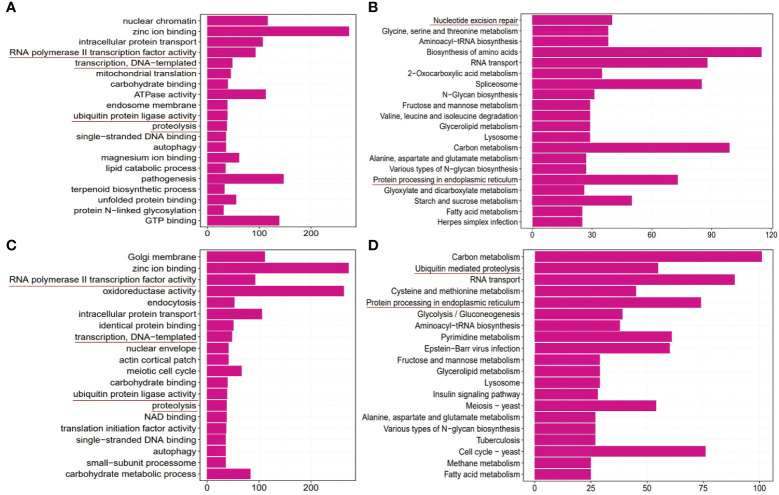
GO and KEGG enrichment of differentially methylated genes (DMGs) in the pyraclostrobin group compared to the control group. **(A)** Top 20 enriched GO terms of 5mC DMGs. **(B)** Top 20 enriched KEGG pathways of 5mC DMGs. **(C)** Top 20 enriched GO terms of the 6mA DMGs. **(D)** Top 20 enriched KEGG pathways of the 6mA DMGs. m5C and m6A DMGs were mainly associated with RNA polymerase II transcription factor, transcription, ubiquitin protein ligase, proteolysis, and nucleotide excision repair and protein processing in the ER. These critical terms were highlighted by underlines in the figure.

A total of 665 5mC DMGs and 596 6mA DMGs were subjected to GO and KEGG enrichment analyses, respectively. The enrichment results are shown in **Figure 8**. According to the results of the GO biological process analysis, both 5mC and 6mA DMGs were enriched in RNA polymerase II transcription factor, transcription, ubiquitin protein ligase and proteolysis. KEGG enrichment analysis revealed nucleotide excision repair and protein processing in the ER in 5mC DMGs and ubiquitin-mediated proteolysis and protein processing in the ER in 6mA DMGs. This finding is consistent with the gene expression results.

### Comprehensive analysis of gene expression and DNA methylation

To further elucidate the effect of DNA methylation on gene expression, we compared DEGs with DMGs (5mC/6mA), of which 23 DEGs showed differential 5mC or 6mA methylation, as shown in **Table 6**. Unfortunately, there was no consistent trend between changes in 5mC/6mA and gene expression. Three upregulated genes were hypermethylated at 6mA sites, including *MGG_14014* and *EFMOG00000000060* in the promoter and *MGG_10588* in the exon. Three upregulated genes were hypermethylated at 5mC sites, including *MGG_08519* and *MGG_10848* in the promoter and *MGG_02210* in the exon. Two upregulated genes were hypomethylated at 5mC sites, including *MGG_05584* in the promoter region and *MGG_00261* in the exon. *MGG_14956* was hypomethylated at the 6mA site in the promoter. *MGG_16403* was hypermethylated at the 5mC site and hypomethylated at a 6mA site in the promoter region. Three downregulated genes were hypermethylated at the 5mC site, with *MGG_07000* and *MGG_09107* hypermethylated in the promoter region and *MGG_11779* in the exon. *MGG_09822* and *MGG_03921* were hypomethylated at a 5mC site in the promoter. *MGG_08486* and *MGG_07605* were hypermethylated at a 6mA site in the promoter. Two downregulated genes, *MGG_08315* and *MGG_00225*, were hypomethylated at 6mA in the promoter region. KEGG annotation revealed that the upregulated genes MGG_14014, MGG_00261 and MGG_10588 were involved in protein processing in the ER, ubiquitin-mediated proteolysis and RNA transport, respectively. The downregulated gene MGG_07000 participated in the glutathione metabolism process. These results suggest that 5mC and 6mA DNA methylation may participate in the stress response by affecting gene expression. However, other genes were not annotated to a pathway. Of course, there were also many genes that showed changes in methylation levels but nonsignificant changes in gene expression (data not shown), suggesting that the connectivity between DNA methylation and gene expression is complex.

**Table 6:** Differentially expressed genes related to methylation changes under pyraclostrobin stress.

Gene name	log2FC	P adj	Gene function annotation	Methylation Change		KEGG pathway
				5mC	6mA	
MGG_01896	2.98	6.72E-09	Isonitrile hydratase	hypo and hyper in P1-2kb	—	—
MGG_14014	2.12	1.20E-07	Ubiquitin homeostasis protein lub1	—	hyper in P2-3kb	Protein processing in ER (ko04141)
MGG_05584	2.01	0.00572	—	hypo in P1-2kb	—	—
MGG_16403	1.74	0.01641	—	hyper in P2-3kb	hypo in P2-3kb	—
MGG_08519	1.73	0.02174	Oxidoreductase sirO	hyper in P2-3kb	—	—
MGG_02210	1.72	0.03529	Vanadium chloroperoxidase	hyper in exon	—	—
MGG_14956	1.64	0.00688	NADH-cytochrome b5 reductase 1	—	hypo in P2-3kb	—
MGG_10848	1.63	0.00278	—	hyper in P0-1kb	—	—
MGG_05892	1.58	0.00442	—	hypo in P1-2kb and hyper in P2-3kb	—	—
EFMOG00000000060	1.55	0.00829	—	—	hyper in P0-1kb	—
MGG_00261	1.30	0.00289	Beta-TrCP	hypo in exon	—	Ubiquitin mediated proteolysis (ko04120)
MGG_10588	1.00	0.04047	Ser-Thr kinase receptor-associated protein	—	hyper in exon	RNA transport (ko03013)
MGG_08315	-5.15	0.03844	1-phosphatidylinositol 4,5-bisphosphate phosphodiesterase 1	—	hypo in P1-2kb	—
MGG_13430	-3.29	0.01136	—	—	hyper in P1-2kb and hypo in P2-3kb	—
MGG_09107	-2.92	0.04551	—	hyper in P0-1-2kb	hyper in P1-2kb and hypo in P2-3kb	—
MGG_00225	-2.49	0.02539	—	—	hypo in P0-1kb	—
MGG_11779	-2.37	0.00027	NFX1-type zinc finger-containing protein 1	hyper in exon	—	—
MGG_08486	-2.24	0.00167	Beta-lactamase-like protein 2	—	hyper in exon and hyper in P1-2kb	—
MGG_16038	-1.88	0.00781	—	hyper in P1-2kb and hypo in P2-3kb	—	—
MGG_09822	-1.63	0.04787	—	hypo in P2-3kb	—	—
MGG_07000	-1.54	0.00494	Ribonucleoside-diphosphate reductase subunit M1	hyper in P1-2-3kb	—	Glutathione metabolism (ko00480)
MGG_07605	-1.52	0.02874	—	—	hyper in P1-2kb	—
MGG_03921	-1.46	0.00781	Momilactone A synthase	hypo in P2-3kb	—	—

## Discussion

*M. oryzae* has been reported to have high pathogenic variation with respect to host range and variety specificity. Pathogenic variation is the main reason why rice blast fungus breaks through the resistance barrier of rice plants or makes fungicides ineffective. *M. oryzae* has evolved sophisticated strategies to attach and subsequently infect its hosts, processes that often involve unique epigenetic changes. Clarifying the metabolism and molecular changes associated with fungicide action is beneficial for understanding the mechanism of resistance of rice blast fungus to drugs, as well as the requisite for the development of effective disease control strategies. In this work, RNA-Seq was used to analyze genome-wide changes in gene expression in response to the popular fungicide pyraclostrobin, and several critical molecules were shown to play important roles in this process.

Chaperones are central to homeostasis in eukaryotic cells and play essential roles in protein quality control in the ER and in ER-associated degradation (Nishikawa et al., 2005; Hartl et al., 2011). Hsp70 is considered a sentinel chaperone that plays an essential role in aberrant protein degradation in the ubiquitin−proteasome system by cooperating with other cellular chaperones to form dynamic and functionally versatile complexes (Fernandez-Fernandez et al., 2017; Rosenzweig et al., 2019). For example, CHIP, a cochaperone of Hsp70, competes for binding to the C-terminus of Hsp70 and ubiquitinates Hsp70-bound substrates, thereby directing the substrates to the proteasome for degradation (Stankiewicz et al., 2010; Rosenzweig et al., 2019). These chaperones cooperate with cellular degradation machinery to guard cells from the deleterious effects of various proteotoxic stresses. Although the functions of the identified DEGs are only recognized at the level of integrated methylome and transcriptome analysis in this study, it is well known that when organisms face large-scale gene damage, SOS repair is initiated, which inevitably leads to many mutations and the generation of many abnormal functional proteins. These abnormal proteins need to be degraded through ER-associated degradation and ubiquitin-mediated proteolysis pathways. In this study, the expression of genes related to these two pathways increased, which is beneficial for cell survival because the accumulation of abnormal proteins is toxic to organisms. If certain mutations allow the organism to survive, then these mutations will be passed on, and the organism will become resistant to the drug. Therefore, the susceptibility of *M. oryzae* to developing drug resistance may be related to its strong ability to degrade abnormal proteins. This prevents cells that have favorable mutations from dying due to the accumulated toxicity of abnormal proteins.

Ribonucleotide reductases, including tryparedoxin and trypanothione, can catalyze the biosynthesis of deoxyribonucleotides from the corresponding ribonucleotides using thioredoxin as H donors (**Figure 4**) (Krauth-Siegel et al., 2003; Comini et al., 2007). The downregulation of *RRM1* expression contributes to high concentrations of trypanothione and tryparedoxin. It has been reported that trypanothione and tryparedoxin are involved in diverse cellular functions, including maintenance of thiol redox activity, oxidant defenses, defense against xenobiotics, ascorbate homeostasis, sequestration of heavy metals, drug resistance and modulation of the host immune response (Fairlamb and Cerami, 1992; Schmidt and Krauth-Siegel, 2002; Comini et al., 2007; Suman et al., 2018; Gonzalez-Chavez et al., 2019). Moreover, ribonucleotide reductase is responsible for the *de novo* conversion of ribonucleoside diphosphates to deoxyribonucleoside diphosphates, providing the precursors necessary for DNA synthesis. The reduction in enzyme activity inhibited DNA replication and repair, decreasing the fidelity of the DNA-coding genetic information, consistent with the inhibition of DNA replication and repair, as shown in **Table 3**.

Cardiolipin is one of the main phospholipids constituting the inner mitochondrial membrane, improving the fluidity of the inner mitochondrial membrane and facilitating the lateral diffusion of respiratory chain complexes in the membrane lipid bilayer (Houtkooper and Vaz, 2008; Paradies et al., 2014). Moreover, cardiolipin is involved in the formation of all enzyme complexes of the respiratory chain, including complexes I, II, III, IV and V and flexible cytochrome c (Fry and Green, 1981; Eble et al., 1990; Robinson et al., 1990; Ott et al., 2002; Yankovskaya et al., 2003). It has been shown that abnormal cardiolipin can dysregulate respiratory chain complex I and mitochondrial quality control (Anzmann et al., 2021). Studies have also revealed that cardiolipin and phosphatidic acid control mitochondrial division and fusion and coordinate the balance between these dynamic processes (Kameoka et al., 2018). Therefore, inhibition of the expression of the *AYR1*, *PLD1* and *CRLS* genes not only disrupts the structure of the mitochondrial inner membrane and electron transport but also leads to an imbalance of phosphatidic acid and cardiolipin, which interferes with mitochondrial fusion and division. The dynamic balance of these two processes is vital for maintaining the functional competence and quality of mitochondria (Youle and van der Bliek, 2012; Ban-Ishihara et al., 2013; Roy et al., 2015; Shirihai et al., 2015).

An increase in alternative splicing events will generate multiple transcripts from a single pre-mRNA, which contributes to the gain or loss of some protein functional domains. This produces protein isoforms that respond to the demands associated with pathogenicity and environmental pressures such as fungicides in fungi as shown by Gehrmann et al. (2016) and Fang et al. (2020). Several gene knockout experiments have confirmed that alternative splicing, which likely generates protein isoforms, is directly involved in the resistance mechanism in *M. oryzae*. For example, the removal of the *MoHMT1* gene, which causes genome‐wide alternative splicing, resulted in increased pathogenicity in *M. oryzae* (Li et al., 2020). Alternative splicing of *MoPTEN* contributes to conidium and appressorium development and invasive hyphal growth of *M. oryzae* in plant cells (Wang et al., 2021). Alternatively spliced SMN orthologs in *M. oryzae* are required for stress resistance and disease development (Liang et al., 2015). Deletion of the splicing factor MoSrp1 verified that alternative splicing participated in mycelial growth, conidiation, and virulence in *M. oryzae* (Shi et al., 2022).

In this study, the expression of several cell cycle-related genes was downregulated or alternatively spliced. Mei2 is considered an RNA-binding protein that can form a complex with a specific RNA species to promote meiosis (Yamamoto, 1996). One study showed that *mei2* gene expression is regulated by cAMP (Watanabe et al., 1988). Moreover, cAMP is generated from ATP, and this process is blocked by pyraclostrobin. This may be one of the reasons for the decreased expression of *mei2*. Additionally, *mcm7* (MGG_09300) and *ANAPC1* (MGG_03314), two other meiosis-related genes, were downregulated 2.17-fold and 1.46-fold, respectively. MCM7 is a DNA replication licensing factor that is involved in the initiation of replication by loading onto DNA replication origins (Cvetic and Walter, 2006; Evrin et al., 2014). ANAPC1 is one of the subunits of the anaphase-promoting complex (APC), which is an E3 ubiquitin ligase that targets cell cycle regulatory proteins for degradation by the proteasome and functions in cell cycle transition (Castro et al., 2005). These results showed that pyraclostrobin may disrupt cell cycle progression by inhibiting cell cycle-related proteins. MGG_04006 is a Rho-GAP domain-containing protein. Ye et al. (2014) reported that there are eight putative Rho GAP proteins in *M. oryzae*. Some Rho GAP proteins, such as MoRga1 and MoLrg1, may play important roles in vegetative growth, conidiation, conidial morphology, appressorium formation and pathogenicity. However, MGG_04006 did not affect fungal development of virulence. Nevertheless, exon skipping occurred in this gene under pyraclostrobin stress, suggesting that it may be needed for fungal survival. However, further research is needed to study the function of this gene.

Emerging evidence has shown that DNA methylation in transcribed regions is involved in the regulation of alternative splicing through two possible mechanisms. First, DNA methylation modulates the elongation rate of RNA polymerase II via CCCTC-binding factor (CTCF) and methyl-CpG binding protein 2 (MeCP2), allowing some weak splicing signals to be identified as involved in splicing (Shukla et al., 2011; Maunakea et al., 2013). Second, DNA methylation-dependent heterochromatin protein 1 (HP1) can recruit splicing factors to alternative exons that contribute to alternative splicing (Yearim et al., 2015). Further study confirmed that DNA methylation of exon-encoding regions is directly involved in the regulation of alternative splicing (Shayevitch et al., 2018). However, additional underlying mechanisms remain to be elucidated (Lev Maor et al., 2015).

Additionally, the distributions of 5mC and m6A were negatively correlated. In regions with high levels of 5mC, the 6mA level was low, and in regions with low levels of 5mC, the 6mA level was high. A negative correlation between the distributions of 5mC and 6mA in diverse fungi has been reported by Mondo et al. (2017). After pyraclostrobin treatment, the levels of both 5mC and 6mA increased, indicating that pyraclostrobin treatment can increase the methylation rate in the gene body and 2 kb upstream and 2 kb downstream flanking regions. The methylation sites alter the binding of certain proteins, affecting the efficiency of RNA polymerase or the binding of splicing factors, thereby affecting transcription and alternative splicing. Studies have shown that 5mC and 6mA in the gene body are associated with enhanced gene expression (Arechederra et al., 2018; Zhang et al., 2018). Yang et al. (2014) reported that there is a causal relationship between C methylation in gene body and gene expression. 6mA has been reported to be associated with active genes, especially RNA polymerase II-transcribed genes (Mondo et al., 2017; Wang et al., 2017). Further studies suggested that the main function of gene body methylation is not to modulate expression during development or respond to the environment but to stabilize gene expression by preventing aberrant transcription from internal cryptic promoters and enhance splicing efficiency to reduce expression variability by excluding the histone variant H2A.Z (Kim and Zilberman, 2014; Bewick and Schmitz, 2017; Zilberman, 2017).

Although DNA methylation can change the functional state of regulatory regions, the connectivity between DNA methylation and gene expression is complex and poorly understood (Schubeler, 2015; Angermueller et al., 2016). Li et al. (2011) showed that 5mC in the promoter of *Pib* plays a positive role in inducing the expression of *Pib* in *M. grisea*. Partial demethylation by 5-azacytidine treatment reduced *Pib* expression and compromised blast disease resistance. Singh and Vinod (2020) also reported that only a small proportion of differentially hypermethylated genes with 5mC showed downregulated expression. These results suggest that aberrant DNA methylation may play both a positive and negative role in regulating gene expression (Zhu et al., 2016). This study is helpful for understanding the adaptation and variation mechanisms of rice blast fungus, and promoting the control of rice blast disease.

## Conclusion

Under pyraclostrobin stress, proteolysis, glutathione metabolism, and alternative splicing were enhanced in *M. oryzae*, while DNA replication, DNA damage repair, and lipid metabolism were inhibited. These changes may be related to the elevated levels of 5mC and 6mA in the gene body. This work enriches the understanding for potential mechanism of quinone fungicides resistance. Meanwhile, it reflects the complexity of the adaptation mechanism of *M. oryzae* to quinone fungicides and the necessity for ongoing research in this field.

## Data availability statement

Both transcriptome and methylome data presented in the study have been deposited in the BioProject database of NCBI. The accession number is PRJNA1096937 (URL: http://www.ncbi.nlm.nih.gov/bioproject/1096937).

## Author contributions

SF: Data curation, Writing – original draft, Formal analysis, Visualization. HW: Data curation, Writing – original draft. KQ: Data curation, Writing – original draft. YP: Formal analysis, Writing – original draft. CL: Data curation, Writing – original draft. XL: Conceptualization, Writing – review & editing.
