# Supplementary material for: The fungicide pyraclostrobin affects gene expression by altering the DNA methylation pattern in Magnaporthe oryzae
Source: Front Plant Sci. 2024 Apr 30;15:1391900. doi: 10.3389/fpls.2024.1391900 (PMC11091397; doi:10.3389/fpls.2024.1391900)
Supplement: Supplementary file 1 [file Table_1.docx]

| Concentration  (μg/L) | Colony diameter  (cm) | Inhibition rate  (%) | Colony growth figures |
| --- | --- | --- | --- |
| 0.0 | 8.40±0.00 | 0 | 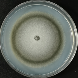 |
| 0.1 | 8.38±0.05 | 0.32 | 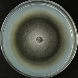 |
| 0.5 | 8.15±0.21 | 3.16 | 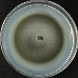 |
| 1.0 | 7.96±0.17 | 5.54 | 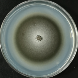 |
| 5.0 | 4.30±0.38 | 51.90 | 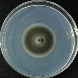 |
| 10.0 | 3.73±0.23 | 59.18 | 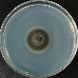 |
| 100.0 | 0.90±0.00 | 94.92 | 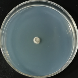 |
| 1000.0 | 0.50±0.00 | 100 | 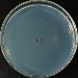 |

Supplemental table 1 Inhibition of hyphal growth by different concentrations of pyraclostrobin
